# Development of a technical assistance framework for building organizational capacity of health programs in resource-limited settings

**DOI:** 10.1186/1472-6963-14-399

**Published:** 2014-09-17

**Authors:** E Michael Reyes, Anjali Sharma, Kate K Thomas, Chuck Kuehn, José Rafael Morales

**Affiliations:** International Training and Education Center for Health, University of California San Francisco, 50 Beale Street, Suite 1300, San Francisco, CA USA; International Training and Education Center for Health, University of Washington, 901 Boren Avenue, Seattle, WA USA; Liverpool Associates in Tropical Health, Liverpool School of Tropical Medicine, Pembroke Place, Liverpool, UK; Global HIV/AIDS Program, HIV/AIDS Bureau, Health Resources and Services Administration, 5600 Fishers Lane, Rockville, MD USA

**Keywords:** HIV, Health system strengthening, PEPFAR, Technical assistance, ART, Sustainability, Capacity building

## Abstract

**Background:**

Little information exists on the technical assistance needs of local indigenous organizations charged with managing HIV care and treatment programs funded by the US President’s Emergency Plan for AIDS Relief (PEPFAR). This paper describes the methods used to adapt the Primary Care Assessment Tool (PCAT) framework, which has successfully strengthened HIV primary care services in the US, into one that could strengthen the capacity of local partners to deliver priority health programs in resource-constrained settings by identifying their specific technical assistance needs.

**Methods:**

Qualitative methods and inductive reasoning approaches were used to conceptualize and adapt the new Clinical Assessment for Systems Strengthening (ClASS) framework. Stakeholder interviews, comparisons of existing assessment tools, and a pilot test helped determine the overall ClASS framework for use in low-resource settings. The framework was further refined one year post-ClASS implementation.

**Results:**

Stakeholder interviews, assessment of existing tools, a pilot process and the one-year post- implementation assessment informed the adaptation of the ClASS framework for assessing and strengthening technical and managerial capacities of health programs at three levels: international partner, local indigenous partner, and local partner treatment facility. The PCAT focus on organizational strengths and systems strengthening was retained and implemented in the ClASS framework and approach. A modular format was chosen to allow the use of administrative, fiscal and clinical modules in any combination and to insert new modules as needed by programs. The pilot led to refined pre-visit planning, informed review team composition, increased visit duration, and restructured modules. A web-based toolkit was developed to capture three years of experiential learning; this kit can also be used for independent implementation of the ClASS framework.

**Conclusions:**

A systematic adaptation process has produced a qualitative framework that can inform implementation strategies in support of country led HIV care and treatment programs. The framework, as a well-received iterative process focused on technical assistance, may have broader utility in other global programs.

## Background

As part of the initial United States President’s Emergency Plan for AIDS Relief (PEPFAR Phase I), the United States Department of Health and Human Services (US DHHS), through the Health Resources and Services Administration (HRSA) and Centers for Disease Control and Prevention (CDC), funded four implementing partners to rapidly scale up HIV care and treatment in countries carrying a high burden of HIV [1]. These four implementing partners (Columbia University, Elizabeth Glaser Pediatric AIDS Foundation, Catholic Relief Services/AIDSRelief and Harvard University) received United States Government (USG) funding to manage and deliver antiretroviral therapy (ART) and other HIV-related services in resource-limited settings. As of August 2012, the implementing partners had worked with CDC country offices, ministries of health and local indigenous partners [2] to provide nearly 4.5 million people with ART [3]. As PEPFAR aims shifted from unprecedented scale up of ART care and treatment in PEPFAR Phase I to prioritizing local country ownership of these programs in PEPFAR Phase II [1, 4], it became paramount to assess local partner readiness to fully manage and implement these programs by February 2012 and achieve the goal of delivering care and treatment to six million people by the end of 2013 [5].

The US DHHS agencies, however, did not have a formal or unified assessment strategy to measure the readiness of local partner organizations to absorb and manage these and other priority health programs. HRSA, with a long history of assessing and strengthening domestic HIV primary care programs, adapted their domestic HIV primary care clinic technical assistance assessment tool to the international context [6].

The Primary Care Assessment Tool (PCAT) was initially developed in 1998 as the HRSA’s HIV/AIDS Bureau’s tool for conducting on-site assessments of primary care programs funded under the Ryan White CARE Act. The tool was designed to assess clinical, fiscal, administrative and other health and support services [6]. The hallmark of the PCAT assessment was the use of strengths-based, participatory and non-punitive approaches to develop findings and recommendations for identifying the specific technical assistance needs of the grantees. The goal was to strengthen the quality of, and access to, primary HIV care in the US.

In collaboration with CDC, HRSA initiated a process to adapt the PCAT to resource-limited settings [6–8]. The resulting framework, the Clinical Assessment for Systems Strengthening (ClASS), was jointly developed with HRSA’s implementing partner, the International Training and Education Center for Health (I-TECH), which is a collaboration between the University of Washington and the University of California, San Francisco [7].

In this paper we describe the methods used and the results obtained in adapting the PCAT to develop ClASS as a framework for assessing organizational capacity and providing technical assistance to health programs in resource-limited settings.

## Methods

Adapting HRSA’s existing PCAT framework entailed using qualitative methods to identify themes and categories most likely to be predictive of success when incorporated into a similar model for use in low-resource health settings [9–11]. General inductive approaches also facilitated the development of a framework based on the proven successes of implementing PCAT in the US health care setting [11]. Stakeholder discussion groups and key informant interviews (including additional interviews conducted one year after implementing ClASS), as well as a review of available assessment tools and a pilot-testing process, were used to develop and refine the framework, approach, review team composition, modules and tools, and technical assistance approach and process (Table 1).

**Table 1:** **Methods used to finalize ClASS framework**

	Source	Date range
1	Initial stakeholder discussions	Intermittently throughout 2008
2	Desk review of assessment tools from Africa and Caribbean	June 1, 2008—September 15, 2008
3	Nigeria pilot	July 9, 2009—August 1, 2009
4	Assessment one year post-ClASS implementation	December 16, 2010—January 21, 2011

Adaptation of the PCAT model to the international context considered the five core elements of the PCAT: approach, team composition, modules, provision of technical assistance, and phases of implementation. Approach would determine capacity, technical assistance needs, and the means by which local indigenous organizations would be reached [12, 13]. Determinations regarding team composition would take into account required skillsets and an assessment of influential stakeholders who would be instrumental for successful capacity building [12, 14]. Different models of technical assistance for capacity building were weighed against the PEPFAR constellation of international, local indigenous and HIV service delivery partners [13]. Finally, the practicalities of implementing ClASS in complex and diverse conditions were considered.

### Initial key informant interviews and stakeholder working sessions

Key stakeholders were interviewed to determine the initial approach to the assessment and review team composition, as well as to inform the development of the overall framework, tools and technical assistance approach for organizations working in low-resource settings.

Adaptations to the PCAT approach and team composition were made based on HRSA’s experiential learning in its application in the US. With general input from HRSA and CDC, the adaptation focused on the overall assessment and technical assistance approach, as well as on the composition of assessment teams. Key stakeholders who were interviewed to inform the adaptation of PCAT included a PEPFAR working group and international implementing partners based in the US, Uganda and Tanzania. These stakeholder interviews identified the expertise required from the assessment and review team.

The data collected from stakeholder discussions were used to develop an initial ClASS framework that incorporated a participatory assessment approach supported by assessment modules appropriate for determining and delivering technical assistance in the international health context.

### Desk review of available assessment tools and ClASS module development

HRSA and CDC collected thirteen tools designed to assess the capacity of health programs directly from ten donor and HIV care and treatment implementing partner organizations working in the international arena. There were no formal collaborative agreements; HRSA reviewed the purpose and application of these tools but did not replicate their content and structure. I-TECH received de-identified tools and systematically compared them for format and content of each tool to identify those best suited to the chosen ClASS approach. Each content area was ranked by frequency of inclusion to identify the operational and technical areas considered most important, which allowed an objective external review of organizational technical assistance needs.

### Piloting the ClASS process

A ClASS visit was piloted in Nigeria in 2009. A local partner in Nigeria had been identified as ready to absorb the largest PEPFAR-funded HIV care and treatment program. Though not representative of all local partners, PEPFAR programs or countries, this application of the ClASS framework to assess local partners’ absorptive capacity and ability to maintain delivery of a quality HIV/AIDS program led to many insights applicable to resource constrained settings as later explained. Around this initial ClASS pilot visit, I-TECH conducted unstructured direct observations, document reviews, individual and group interviews with all stakeholders, and collected fifteen self-administered questionnaires from ClASS team members to assess the utility of the ClASS approach. Data from the pilot visit were analyzed using a general inductive approach [11] to recommend improvements to the processes for pre-visit planning and preparation and for the selection, training and coordination of ClASS team members. Additional improvements were also recommended for the ClASS modules and for the itineraries to be used at both local indigenous partner and local partner treatment facility levels. The ClASS framework, approach and tools were accordingly refined before further implementation.

### Stakeholder feedback after one year of implementing the ClASS framework

To further assess the utility of ClASS in the international health setting, an I-TECH consultant conducted thirty- to ninety-minute individual and group telephonic interviews with twenty-six ClASS stakeholder respondents between December 15, 2010 and March 7, 2011. Stakeholders identified by HRSA and implementing partners were asked about their experience with implementing ClASS, participating in ClASS visits, the ClASS processes, modules, its products (reports and action plans), and continuous improvement processes. Data were analyzed using thematic analysis. Emerging themes were iteratively explored and clarified during the interviewing process. Once all interviews were completed, interview notes were read and re-read to identify recurrent themes, which were then compared across stakeholder type and further triangulated with process monitoring data. The resulting information was used to make recommendations on improving the overall ClASS framework, its continuous quality improvement processes, the modules and tools, assessment report writing, and provision of technical assistance [9, 10].

Our manuscript reporting adheres to the journals guidelines for relevance, appropriateness, transparency and soundness of interpretation (RATS) for reporting qualitative studies.

The activities described in this paper did not meet the US federal definition of human subjects research. As such, the University of Washington Human Subjects Division determined that human subjects ethics review and oversight was not required for these activities.

## Results

### The ClASS framework to meet the needs of HIV care and treatment programs in low-resource settings

Stakeholder discussions and interviews, comparison of existing tools, pilot-testing and additional stakeholder feedback one year after ClASS had been implemented, together resulted in the development of the ClASS framework, approach, assessment and review team composition and roles, modules and tools, a model for provision of technical assistance and implementation process [15]. The resulting ClASS toolkit was then made publicly available by HRSA for use by professionals assessing the capacity and technical assistance needs of health organizations in low-resource settings [7].

### The ClASS approach

The ClASS approach was modeled after key elements of the PCAT participatory approach, which was designed to identify capacity gaps and provide the technical assistance needed to address them [6, 16]. Development of the ClASS approach required identifying the differences between domestic and international programs and making appropriate modification for them [17]. Ryan White programs were federally funded by one government, bound to the same standards and guidelines, and implemented through stand-alone primary health units [6, 16]. On the other hand, PEPFAR programs were managed by organizations bound to multiple US federal and private donors; were subject to the laws, standards, and guidelines of various national and local governments; and they could be implemented through facilities ranging from small health posts to large tertiary referral and teaching hospitals [18]. HRSA, eschewing an audit-like assessment, proposed a participatory approach to include key stakeholders such as donors, partners at all levels and, where possible, ministries of health. The approach employed a comprehensive assessment of technical, organizational, and managerial capacity to deliver quality clinical services and manage related funding at three levels of partnership: implementing partner, local indigenous partner, and local partner treatment facility. In addition, the proposed HRSA approach was to offer an external and supportive review of organizational capacity that did not replicate country-led planning, routine assessments, and routine provision of technical assistance.

Based on the results of the pilot in Nigeria, the ClASS framework rested on participatory approaches and authentic engagement to include the use of independent reviewers; a "no surprises" guarantee in formal communication post-ClASS visit; the recognition of the "snap shot" nature of the assessment; and, most importantly, the "spirit" of the mission, which is to assist with capacity development to prepare for transition rather than to find fault [14]. Stakeholder feedback after a year of use indicated that the ClASS approach did not need refinement and that the interactive participatory process and authentic engagement were well-received novel experiences for most international and local partners.

### ClASS team composition

Deliberations with stakeholders revealed factors that warranted consideration while determining the assessment and review team composition, including the scope of work, the size and type of the program, the number of treatment facilities to be visited, language competencies and preferences, and the availability of resources. Given the ClASS participatory approach, the ClASS team included stakeholders, external reviewers, and both stakeholder and external technical assistance providers [19].

The pilot study and stakeholder feedback after one year confirmed that inclusion of all stakeholders in the ClASS review led to mutual understanding of program strengths and constraints and constructive dialogue to enhance organizational capacity. The pilot indicated the need to clarify roles for all team members. The HRSA project officer role as coordinator/liaison was differentiated from that of the team leader as the technical lead and synthesizer of information. The pilot also revealed the leadership challenges posed by having multiple teams assessing different program components at different levels of implementation (international, local, health facility). The pilot study and one-year post-ClASS implementation assessment identified the following as characteristics of effective ClASS assessment and review team members:

1. Subject area expertise and in-depth practical experience to problem solve, generate solutions and provide on-the-spot capacity-building suggestions as part of the assessment process.
2. An understanding of HIV care in the context of the treatment facility visited (for instance guidance on CD4 cut-off for ART initiation).
3. Cultural competence, particularly in the region/country where treatment facilities are visited.
4. Strong communication skills to facilitate constructive discussions on areas that can be improved.
5. Diplomacy and skill in managing/negotiating multiple agendas to meet varied stakeholder interests.
6. Flexibility and organizational skills to adapt the application of the ClASS framework to suit the local operational context and time constraints.

### ClASS modules

The existing PCAT modules were adapted for use by ClASS in international settings through systematic comparisons of thirteen assessment tools that had been used in Africa and the Caribbean to evaluate organizational capacity. The existing tools were variations of numerical rating scales and yielded primarily quantitative, numerical data.

These facility- or organizational-level performance scales were either in the form of checklists with a score for the presence of each item (allowing for comparison between facilities) or had pre-defined scales ranging from zero to five, where zero was the complete absence of desired functionalities (e.g., no access to laboratory tests) and five was complete functionality (e.g., test results received from accredited laboratory with good laboratory practices and provision for external quality assurance tests). These existing tools did not support ClASS’ participatory, comprehensive and non-punitive approach, so were adapted to PCAT’s qualitative approaches to better suit the needs of ClASS.

**Figure 1:**
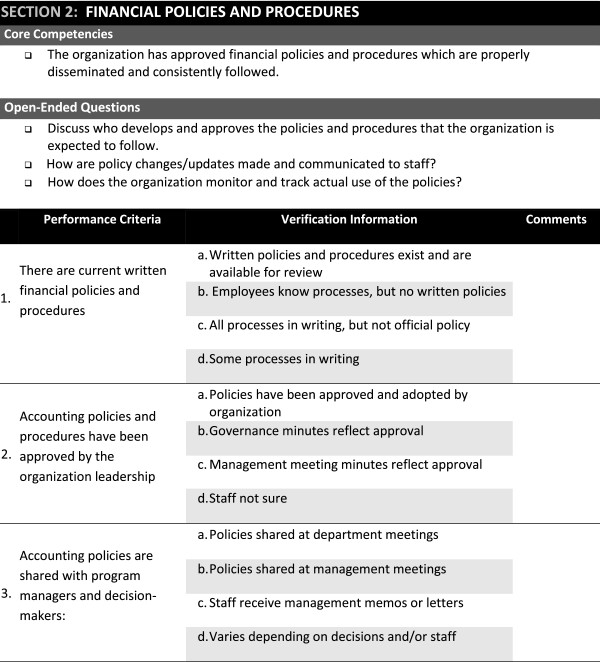
**Snapshot of the Early ClASS Financial Module Illustrating Structure and Content (January 2010).**

The comprehensive review resulted in a synthesis of information into three modules to facilitate systems strengthening activities. The PCAT format was retained for the ClASS modules which listed key questions followed by a verification checklist (see Figure 1). Key questions were developed to elicit discussion on specific focus areas, and the verification checklist was developed to be used as a probe for items not previously discussed. Through a review of clinical, administrative, fiscal and technical competencies at the partner organization, the ClASS modules were designed to yield a comprehensive assessment and/or validation of local partner capacity and readiness for provision of local partner management and support to treatment facilities.

The results of the comparisons yielded twenty-seven standard focus areas, and the pilot revealed the need to include grants management and monitoring as the twenty-eighth focus area (Table 2). A modular format consisting of three modules (administrative, clinical and fiscal) was chosen to assess organizational strengths and technical needs across the twenty-eight standard focus areas.

**Figure 2:**
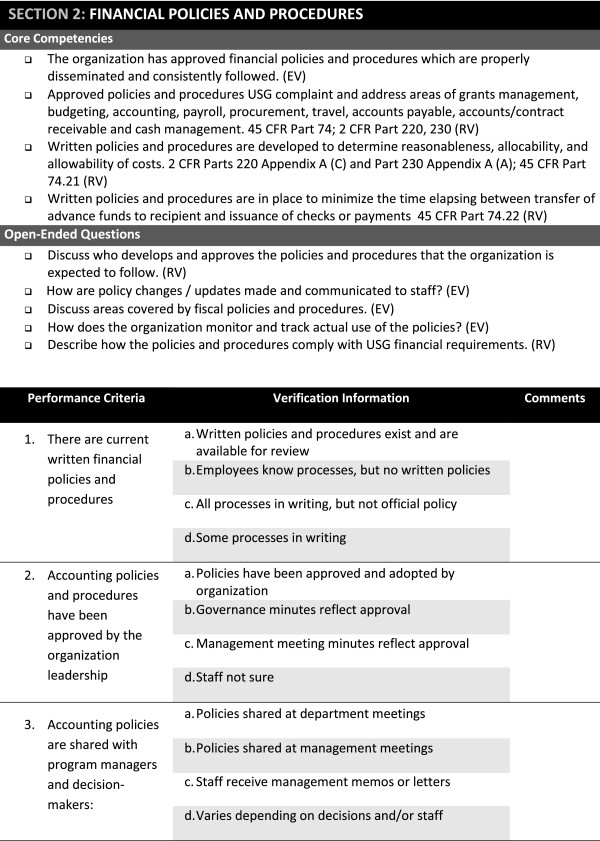
**Snapshot of the Current ClASS Financial Module (December 2012).**

Based on time constraints experienced at the piloted ClASS visit, the modules were redesigned to contain mandatory questions (estimated time: two hours); other important questions to be asked if time permitted (estimated time: four hours); and questions that might be relevant to ask if there was still more time. The feedback at one year post-ClASS implementation made explicit the need to better articulate the standards underlying the key questions and to distinguish those competencies essential for transitioning donor compliance from those that were "best practices" or simple enhancements to current systems. Accordingly, the ClASS modules and tools were further refined (Figure 2).

**Table 2:** **Final ClASS focal areas**

#	Focal areas by module
	**ADMINISTRATIVE**
	***Assesses:*** *Ability to support effective and efficient program implementation*
1	Organization and structure
2	Governance
3	Strategic and short term planning
4	Grants management and program monitoring^†^
5	Human resource management
6	Personnel policies and procedures
7	Clinical personnel issues
8	Licenses and certifications
9	Risk management and liability protection
10	Quality assurance
11	Supply chain management networking
12	Collaboration, linkages
13	Management information systems
	**FISCAL**
	***Assesses:*** *capacity to provide the funded services and manage funding; provide management and support to HIV treatment and care facilities*
14	Income and expenditures
15	Charges and fees
16	Billing and collections
17	Accounting system
18	Accounts payable and cash flow
19	Fixed assets
20	Inventory and purchasing
21	Payroll
22	Revenue
23	Cost allocation
	**CLINICAL**
	***Assesses:*** *integration and quality of care; ability to maintain care post- transition; ability to provide technical support/oversight to healthcare delivery*
24	Facility structure
25	Policies & procedures
26	Project work plan
27	Continuous QI/QA
28	Medical record reviews

^†^Included in original list from the content analysis based on pilot in Nigeria.

### Role of ClASS team in providing technical assistance

The assessment was designed to lead to the provision of technical assistance that would promote health systems strengthening, high quality services and local program ownership. This element of the ClASS framework was informed by the underlying principle that external assistance would be provided for a limited period and that it would build on existing strengths, resources and problem-solving capabilities [13]. Implementing partners were expected to provide technical assistance throughout the entire transition process by strengthening policies and practices, building skills, and nurturing partnerships and networks [13].

The pilot showed the capacity of the ClASS reviewers to serve as instant technical assistants, and it further suggested the value of greater inclusion of the implementing partner in the assessment itself. After a year of ClASS implementation, several improvements were suggested. These included a greater role for ClASS reviewers in differentiating between critical and ideal recommendations, as well as making clear the underlying principles, standards and regulations informing the technical assistance component of ClASS. ClASS reviewers were also asked to provide solutions to suit incremental levels of effort, resources, and time.

Additionally, local partners were asked to increase their role in prioritizing activities to strengthen their health systems. This included identifying external assistance (from the implementing partner or another source) and/or internal assistance (from the reviewers themselves), as long as there was not a potential conflict of interest.

Further recommendations for strengthening the technical assistance component of ClASS also included increasing the role of donors in clarifying, particularly for post-transition purposes, who is responsible for implementing ClASS recommendations and to whom they are accountable. HRSA was asked to provide a list of consultants who could meet the specific technical assistance needs of the organization, should implementing and local partners need to find technical assistance outside their country or region. Provision of continued monitoring and follow-up ClASS visits were also identified as ways to support the program transition to local ownership.

### ClASS processes

Implementation of ClASS was far more complex than that of PCAT because of its international scope and multitude of players. Data obtained from stakeholder interviews resulted in the development of a phased implementation approach [15].

HRSA and I-TECH staff developed several tools and communication methods to assist the assessment teams, the recipients of the ClASS visits, and the other stakeholders during each phase of the ClASS process. These tools were developed, piloted and refined, and were ultimately made available for broad use on the ClASS website [7].

When the ClASS process was evaluated one year post-implementation, stakeholders preferred the revised ClASS report format. One major reason for this is that the resulting report now had a condensed "executive summary" highlighting findings and recommendations germane to both the delivery of high-quality HIV care and treatment and to the transition to local ownership. Inclusion of the matrix of findings and recommendations, which were supported with a template for action planning, was seen as useful for planning, implementation and monitoring by all stakeholders. Stakeholders further suggested that the report structure, like the tools, should be responsive to the purpose of the ClASS visit. It was also recommended that the information contained in the final report should, when possible, be illustrated using tables, graphs, pictures and other visual aids that can help quickly guide the reader through the report.

The ClASS framework allowed for continuous quality improvement through feedback collected about reviewers and ClASS processes. The stakeholders preferred to give in-person feedback at the end of the ClASS visit while observations were still fresh.

## Discussion

In November 2011, in response to progress made and milestones achieved in HIV/AIDS prevention, treatment and care, US Secretary of State Hillary Clinton proposed a vision for an AIDS-free generation [20]. One critical step towards attaining that goal is the progressive enrollment of HIV-infected individuals into antiretroviral therapy programs as a means of reducing HIV transmission [21]. With PEPFAR’s increasing emphasis on transitioning programs to country ownership, the Office of the Global AIDS Coordinator has defined four aspects of country ownership essential for program transition. These are: political ownership/stewardship, institutional ownership, capabilities and accountability [4].

In the absence of an existing USG framework, ClASS was developed to identify and address the technical assistance needs of local indigenous organizations tasked with the management and maintenance of these large programs in order to realize PEPFAR’s goal of country ownership of HIV care and treatment programs. Adapting a previously-employed schema (PCAT), we developed a framework that included plans for approach, assessment team composition, modules and tools, technical assistance model, and phases of implementation. The resulting ClASS methodology has proven to be a useful assessment framework that can be adapted by local governments and donors as reported in a previous publication [22].

Systematic literature reviews conducted from 2003–2013 on capacity building [23–27], innovation [26, 28], and program scale-up [29, 30] demonstrate the strength and utility of frameworks which have long been in use [31]. These documents helped define best practices, i.e., those practices that have consistently shown superior results to other methods or techniques. In many cases, "best" practices readily applicable to individual entities proved too cumbersome, costly or impractical to implement at scale leading to practices most commonly employed in the industry ("industry standard" practices). Also, best practices for building individual organizational capacity differ from those for building capacity of the sector (i.e. the healthcare industry encompassing all players involved in the promotion, restoration and maintenance of health) and sub-sector (i.e. health field such as HIV/AIDS, tertiary care). Table 3 shows how the ClASS framework meets most criteria of best practice even when applied internationally and across varied organization types [31]. These are further discussed below.

**Table 3:** **Capacity assessment framework best practices, industry standards and ClASS practices classification**

Capacity assessment framework best practices, industry standards and ClASS practices classification				
Component		Best practice	Industry standard	ClASS practice classification
A	Purpose and Expected Outcomes	• All parties share an understanding on the purpose of the capacity assessment (define strengths and needs) and how the results will be utilized (develop a capacity building plan).		Best Practice
		• Prior to initiating the assessment process, the funder defines future support available to implement the capacity building plan (available technical assistance, funding, etc.).		
B	Assessment Driver	• Organization identifies the need for an assessment	• Funder defines the need for assessments	Industry Standard
C	Assessment Timing	• Conducted before initiating capacity building activities		Best practice
D	Define what will be assessed	• Holistic assessment of organizational management and programmatic processes and structures.	• Process and structures for:	Best practice
			• Organizational Management	
			• Specific thematic area (finance admin, etc.)	
			• Programmatic Area (Clinical, Community Development, etc.)	
E	Assessment Process	• Participatory approach where the entity buys into the need for an assessment		Best practice
		• Facilitated self-reflection		
F	Facilitation	• External facilitation team	• External facilitator	Best practice
G	Data Collection	• Utilize multiple data collection sources (may include interviews, focus groups, staff/client surveys, document review, observation)	• Interviews and document review	Best practice
H	Assessment Tool	• Select tool best suited to the organization’s needs	• Tool developed by the funder, or use of an existing tool that meets the funder’s needs	Industry standard
		• Adapt tools to address organization’s needs, cultural differences, local context		
I	Tool "measurement"	• Qualitative analysis of assessment criteria	• Semi-Qualitative benchmarks per indicator	Best practice
J	Preparing for the Assessment	All involved parties fully understand and are committed to supporting the process, tools, timeline, and time commitment		Best practice
K	Assessment Results	Used to develop a Capacity Building Plan through a participatory process with the assessed entity to review assessment results, define and prioritize needs		Best practice

HRSA has used the ClASS framework to support the transition of thirteen HRSA/PEPFAR programs from international to local partners. This includes Harvard University’s Nigeria program, the largest of all the HIV care and treatment programs, with funding of approximately $40 million USD at its peak. All international partners supported by HRSA actively built the capacity of local partners by providing the technical assistance they needed to meet the proposed 2012 transition. ClASS helped to focus and accelerate the momentum of their capacity building efforts by identifying local partner and treatment facility-specific technical assistance needs. In response to PEPFAR Phase II guidance, and in some cases aided by the successful implementation of the ClASS framework and tools, HRSA-supported HIV care and treatment programs are transitioning to governments (Rwanda), faith-based organizations (Ethiopia, Guyana, Haiti, Kenya, Tanzania, Uganda, and Zambia), or local non-governmental organizations (Botswana, Nigeria and Tanzania).

The implementation of the ClASS framework can be labor- and cost-intensive, but the well-articulated implementation processes can be easily tailored to suit time and resource constraints. The five components of the ClASS framework are supported by a web portal [7]. As with any toolkit, users are able to use any component of the ClASS framework to meet their specific needs. For instance, organizations that already have assessment modules may adapt the ClASS approach, use ClASS-trained reviewers, and source technical resources from the ClASS website. They may not need to involve such a wide array of stakeholders, reducing levels of effort for preparation and planning.

HRSA has invested in training 18 experts in sub-Saharan Africa on the ClASS framework, particularly on its approach, through didactic and experiential learning under the guidance of veteran ClASS reviewers. The cadre of expert reviewers, based in 8 countries in the Horn, East, West and Southern Africa, contribute knowledge and expertise in various fields, as well as deep experience working with health programs in resource-constrained settings. Accessing this existing wealth of knowledge, experience and cultural competence has enhanced the implementation of the ClASS framework and reduced associated costs. HRSA and I-TECH offer periodic training to keep these reviewers abreast of the latest developments in ClASS and USG requirements. ClASS reviewers can be contacted via I-TECH or the HRSA ClASS website [7].

Frameworks are emerging for capacity building and a variety of transition metrics and tools from PEPFAR are being developed. For now, ClASS is offered as a qualitative framework that is complementary to quantitative ones. Because all ClASS processes and stakeholders lend themselves to capacity building, it is difficult to isolate the specific necessary and sufficient conditions for sustainable capacity building. The very acts of donor engagement and inquiry serve as impetuses to change [32]. The shared modules, with instructions for self-assessment, help focus organizations and the ClASS review. The action plan supports the use of the local partners’ internal resources, and the PEPFAR model ensures that the implementing partners facilitate experiential learning [33]. While PEPFAR guidance for governance and fiscal responsibility does exist, ClASS assessments can be challenging in the absence of locally-relevant standards or requirements. In addition, donor requirements must be reconciled with national health policies, procedures and systems. As was learned in the domestic PCAT experience, the ClASS process presents an opportunity to share best practices across programs that may have a greater relevance to low-resource settings.

The ClASS framework has been adapted for strengthening the oversight provided by in-country CDC and regional ministries of health in Tanzania, training institutions in South Africa, HIV centers in the Ukraine, and the Medical and Nursing Education Partnership Initiatives (MEPI/NEPI) in sub-Saharan Africa. These efforts aim to build capacity to function effectively within different institutional and policy environments. Whether the use of ClASS as a standalone framework yields sustained capacities and demonstrated capability in resource-limited settings should be tested in the coming years.

### Limitations

The ClASS framework is not without challenges. In many cases, participants were skeptical of the usefulness of the ClASS approach because the participatory, strengths-based approach was a sometimes novel concept in the settings in which it has been implemented.

While successfully participatory, the ClASS framework and approach has not been able to consistently engage ministries of health due to their competing priorities and time commitments. To address this limitation and ensure full representation, the comprehensive nature of ClASS sometimes requires going beyond the ministries of health to, for example, the ministries of local government or administration.

Also, the ClASS framework has not included patient and other communities in the review process. This would be an area to further develop, and would provide a mechanism to measure health outcomes resulting from ClASS processes at the patient and community level.

The ClASS modules do not currently include quantitative measures that would allow organizations to rank themselves or track progress to transition readiness, country ownership or sustainability [6]. Creating these metrics can be difficult. There are no definitions of ideal organizations, organizational frameworks or necessary and sufficient conditions that foster strong, sustainable organizations. While some of the assessment tools reviewed before deciding on the ClASS modules do have performance scales, the hierarchy of functionality inherent in those scales is not necessarily validated.

## Conclusions

Determining the technical assistance needs of HIV care and treatment programs in resource-limited settings can inform donor agency strategies for ensuring successful transition to local health program ownership. Providing and disseminating this information is critical if the goal of country ownership is to be realized. The ClASS process, as an iterative process focused on developing technical assistance activities to support local indigenous partners in absorbing programs previously funded by USG, may have broader utility for other multilateral initiatives. ClASS has been well received by in-country partners and is perceived as respectful in its implementation and effective in its results.

The PEPFAR mandate to transition to local partners is laudable. As donor, national and disease priorities shift, there is a need to recognize local indigenous organizations as true owners of their health programs. As such they are deserving of resources that include ongoing technical assistance to assure quality outcomes that successfully sustain vital ART care and treatment programs.

## Abbreviations

ART: Antiretroviral therapy
CDC: U.S. Centers for Disease Control and Prevention
ClASS: Clinical assessment for systems strengthening
DHHS: Department of Health and Human Services
HRSA: U.S. Health Resources and Services Administration
I-TECH: International Training and Education Center for Health
PCAT: Primary care assessment tool
PEPFAR: U.S. President’s Emergency Plan for AIDS Relief.

## References

[1] El-Sadr WM, Holmes CB, Mugyenyi P, Thirumurthy H, Ellerbrock T, Ferris R, Sanne I, Asiimwe A, Hirnschall G, Nkambule RN, Stabinsky L, Affrunti M, Teaside C, Zulu I, Whiteside A (2012). Scale-up of HIV treatment through PEPFAR: a historic public health achievement. J Acquir Immune Defic Syndr.

[2] **Fiscal Year 2012 Country Operational Plan (COP) Guidance** http://www.pepfar.gov/documents/organization/169694.pdf

[3] **Using science to save lives: Latest PEPFAR results** http://www.pepfar.gov/documents/organization/187770.pdf

[4] **Fiscal Year 2013 Country Operational Plan (COP) Guidance** http://www.pepfar.gov/documents/organization/198957.pdf

[5] Government Accountability Office (US) (2010). President’s Emergency Plan for AIDS Relief: Efforts to Align Programs With Partner countries’ HIV/AIDS Strategies and Promote Partner Country Ownership.

[6] Institute of Medicine Committee on the Ryan White CARE Act (2004). Measuring What Matters: Allocation, Planning, and Quality Assessment for the Ryan White CARE Act.

[7] **Clinical Assessment for Systems Strengthening** http://www.classtoolkit.org

[8] **A Successful Framework for Assuring the Quality and Sustainability of US Government-Supported HIV Care and Treatment Programs in Resource-Limited Settings** http://www.classtoolkit.org/class-monograph

[9] Green J, Thorogood N (2004). Qualitative Methods for Health Research.

[10] Bradley EH, Curry LA, Devers KJ (2007). Qualitative data analysis for health services research: developing taxonomy, themes, and theory. Health Serv Res.

[11] Thomas DR (2006). A general inductive approach for analyzing qualitative evaluation data. Am J Eval.

[12] Russ-Eft DF (2009). Evaluation in Organizations: A Systematic Approach to Enhancing Learning, Performance, and Change.

[13] Crisp BR, Swerissen H, Duckett SJ (2000). Four approaches to capacity building in health: consequences for measurement and accountability. Health Promot Int.

[14] Perla RJ, Bradbury E, Gunther-Murphy C (2013). Large-scale improvement initiatives in healthcare: a scan of the literature. J Healthc Qual.

[15] **Clinical Assessment for Systems Strengthening: ClASS Overview/The Class Process** http://www.classtoolkit.org/overview

[16] Linsk NL, Bruce D, Schechtman B, Warnecke R, Tunney K, Bass M (2005). Development and implementation of a quality improvement program for Ryan White Title I care services using a stakeholder-based model. AIDS Patient Care STDS.

[17] Svornos T, Mate KS (2011). Evaluating large-scale health programmes at a district level in resource-limited countries. Bull World Health Organ.

[18] Kanki P, Kakkattil P, Simao M (2012). Scaling up HIV treatment and prevention through national responses and innovative leadership. J Acquir Immune Defic Syndr.

[19] **Clinical assessment for systems strengthening: assessment preparation document** http://www.classtoolkit.org/tools/assessment-preparation-documents.

[20] Goosby E (2012). The President’s emergency plan for AIDS relief: marshalling all tools at our disposal toward an AIDS-free generation. Health Aff.

[21] Cohen MS, Holmes C, Padian N, Wolf M, Hirnschall G, Lo YR, Goosby E (2012). HIV treatment as prevention: how scientific discovery occurred and translated rapidly into policy for the global response. Health Aff.

[22] Sharma A, Chiliade P, Reyes EM, Thomas KK, Collens SR, Morales JR (2013). Building sustainable organizational capacity to deliver HIV programs in resource-constrained settings: stakeholder perspectives. Glob Health Action.

[23] Merion S, de los Rios Carmenado I, de los Rios Carmenado S (2012). Capacity building in development projects. Procedia - Social Behavior Sciences.

[24] Baser H, Morgan P, Bolger J, Brinkerhoff D, Land A, Taschereau S, Watson D, Zinke J (2008). European Centre for Development Policy Management.

[25] Program UND (2005). A Brief Review of 20 Tools to Assess Capacity.

[26] Johnson K, Hays C, Center H, Daley C (2004). Building capacity and sustainable prevention innovations: a sustainability planning model. Eval Program Plann.

[27] Doherty S, Mayer S (2003). Results of an Inquiry Into Capacity Building Programs for Nonprofit Organizations.

[28] Chaudoir S, Dugan A, Barr C (2013). Measuring factors affecting implementation of health innovations: a systematic of structural, organizational, provider, patient and innovation level measures. Implement Sci.

[29] Subramanian S, Naimoli J, Matsubayashi T, Peters D (2011). Do we have the right models for scaling up health services to achieve the Millennium Development Goals?. BMC Health Serv Res.

[30] World Health Organization (2007). Scaling up Health Service Delivery: From Pilot Innovations to Policies and Programs.

[31] MacLeod P (2013). Clinical Assessment for Systems Strengthening (ClASS) Model and Methodology Review: Findings and Recommendations.

[32] Bushe GR, Boje D, Burnes B, Hassard J (2011). Appreciative Inquiry: Theory and Critique. The Routledge Companion to Organizational Change.

[33] Wandersman A, Chien VH, Katz J (2012). Toward an evidence-based system for innovation support for implementing innovations with quality: tools, training, technical assistance, and quality assurance/quality improvement. Am J Community Psychol.

[CR34] The pre-publication history for this paper can be accessed here:http://www.biomedcentral.com/1472-6963/14/399/prepub

